# Business Building

**Published:** 1910-01-15

**Authors:** William H. Trueman

**Affiliations:** Philadelphia, Pa.


### BUSINESS BUILDING.

BY WILLIAM H. TRUEMAN, D.D.S., PHILADELPHIA, PA.

Business building is in the air. Not only in dentistry but in all lines of work, by which an honest living may be made. The suggestion in your editorial comment on page 552 of the November Register, is in line with much that has recently appeared in dental journals under a like heading. The commercialism involved is rather rasping to ethical ears, nevertheless, the prosy fact of food wanted, the need of lodging, clothing and provision against the time when advancing years makes a grasshopper seem a burden, will intrude to break the rhyme of charming poetry, and mar by discordant notes the enrapturing melody of "how we would like things to be." How to get more business, and how to make business return a larger profit, is to-day the vexed question over which many minds are pondering. In the commercial world, some have solved it to their own satisfaction by combining together to economize production and to open new and to control existing markets. This is called on the one side, thrift, energy, business enterprise, philanthropy, and other endearing names. It has, indeed, in many ways advanced public interests, made remunerative employment for many, added to the comforts of life, and made many waste places blossom as with roses. It has opened new avenues to commerce and to civilization, and in a thousand and one ways has proved a public blessing. However, in doing all this, it has caused some to suffer, and inasmuch as the more visible profits have landed in but a comparatively few hands, those bringing it about have been stigmatized as greedy, soulless corporations and conscienceless trusts.

Some of your suggestions, Mr. Editor, are a like combination of good and evil. It is undoubtedly true that in a town of three thousand inhabitants are many needing dental work and suffering for the want of it, many, perhaps, who are well able to properly remunerate a well qualified dentist. It was an excellent idea that of the wise old mouse who suggested belling the cat; but, how to do it was a vexed problem. It is an excellent idea to bring to the notice of the public the evils arising from neglected teeth other than those announced by a raging toothache, but, how shall it be done? Nicely written, readable books and booklets, pages and paragraphs in the newspapers, and lectures now and again, are the oldest forms of dental literature and dental instruction extant, we may trace them back to the year one. That they are still called for seems evidence of their ineffectiveness. The catchy business advertisement seems far more effective, if it is true, that the advertising dentist is usually a busy dentist. It is true, undoubtedly, that quite a number of the more useful improvements in dental art have been introduced to the public and to the profession by the advertising members of the calling. Who introduced amalgam? Who brought to public notice the decided advantages of porcelain artificial teeth over the old time, disgustingly filthy bone dentures? Who made crown and bridgework popular? How was porcelain inlays introduced to the public? These ideas were "old as the hills," well known to the well informed, but were dorment until enterprising dentists took hold of them and by public advertisements made them known to the public and by so doing forced the ethical members of the profession to take note of them. Of course it is a shock to our ethical members to know this, but, nevertheless it is true. How we like to malign the natty Frenchmen who introduced the much abused amalgam to the public of New York some eighty years ago, but what a godsend it has been to the public and to the profession! Who taught our New York friends that a tooth riddled by caries could be made to do comfortable service for years by an easily placed plastic metal filling? Was it not those same natty Frenchmen, who our honorable professional forefathers proudly claim the honor of having hounded out of the town? What they could not do the Frenchmen did. They demonstrated this decided advance in tooth saving, and created a demand for it, then our ethical forefathers took note and followed their lead. Later they did better than their teachers. By a like process was porcelain dentures forced upon the profession. Our Detroit friend took hold of porcelain fillings when they were known to only a few, and by his artistic skill, his mechanical ingenuity, and his persistent presentation of their merits to the public and the profession forced them into prominence as an artistic tooth saving device. He was not ethical, but he had sufficient public spirit, and sufficient confidence in the thing he advocated to brave the odium, and deserves the credit of making porcelain fillings popular with the public and profitable to the profession. Crown and bridgework was on record more than a thousand years, known to all, but practiced by but few until Dr. Shepherd plastered the whole country side with handbills and gaudy painted signs, and deluged the mails with circulars and pamphlets, and presto! as a result of all this, are not dentists the world over raking in the golden ducats by supplying the public with crown and bridgework? Unwilling as we all are to acknowledge it, truth compels us to admit that the advertising methods are the methods that bring results. We condemn the advertiser as thoughtlessly as some say their prayers, nevertheless, there is not in the world to-day a community, nor a dentist, that has not been bettered by what they have done. You are right, undoubtedly, that there is far more work to do in that little town than the complaining dentist gets; but, how is he to get it? Unfortunately, as a rule, business ability and professional skill and integrity are seldom found in the same individual. The ideal professional dentist starves, while the stigmatized and successful advertiser lives in clover, all the while ruining the patient's precious teeth. Our complaining friend would hardly like to go through that little community hunting orthodontia cases to help his bank account, nor yet to stand at his front door asking passers-by to step in and have their teeth scrubbed. To do either would most likely prove his utter ruin.

Your suggestion to add to his charges is excellent in theory. So could the grocer add to his profits by charging a few cents a pound more for his sugar, and the dental depot would make a better showing to its managers by charging a dime more per tooth. This as you state, would be clear profit. Would it be wise? For my own part I have preferred the nimble, oft repeated six pence to the slow moving infrequent pound, and am quite satisfied with the result.

I fear, and fear very much, the outcome of a practice that permits a dentist to save but one-twentieth of his in-taking. It is not so much the smallness of his in-taking as the largeness of his out-go. Two-thousand dollars a year, in a country town, may not be small, but the nineteen-hundred dollar out-go seems quite out of proportion, and it is high time to take stock and see what can be done to mend matters. Are there not some nearby towns in which a branch office could be advantageously established? Prosperity is by far the most economical and the most effective ethical advertisement. The busy, pushing, prosperous man or the man who seems to be so, is the man of all others the world wants to help. A few years ago, Harper's Bazar invited the wives of men with moderate income to write how that income was used, and the published statements of how incomes of from $800 to $2,500 were spent proved suggestive reading. So far as the published accounts showed they came from good, wisely economical housekeepers, and yet the amount available for medical and dental attention was exceedingly small. Now, in the United States there are vastly more breadwinners, above the laboring and mechanic class, whose incomes are less than $2,000, than there are above. Clerks, teachers, men in public office, and many professional men, with average yearly incomes of less than

$2,000. These small incomes do not allow much for dental work. Tooth cleaning at the rate of five dollars each, for a family of eight, would make a big hole in the allowance. It will be well for the "charge more" advocates to take note of this. A wider field, rather than a higher fee, is by far the safest proposition.

Dentistry does not stand as well with the community as does medicine because heretofore it has been all commercialism—physicians and surgeons have shown their public spirit by working for the public good. They have manned and maintained hospitals, erected and stocked medical libraries, their library buildings are ornaments to many cities. They look after the public health in various ways, and ways that are not "money getters" to themselves. A threatened epidemic of small pox in Philadelphia was nipped in the bud by a squad of physicians under police protection making a midnight raid covering several blocks, routing men, women and children out of their beds and compelling them to be vaccinated. (It was illegal, but the only way to do effective work in that section). A medical society started a movement and kept dogging at it until we have filtered water, they have demanded improvements in sewers, cleaner streets, etc., all this making their services less necessary, depriving them of thousands of dollars in fees. Their prevention tends to impoverish the profession while doing a world of good to the present and to coming generations. Dentists have done none of these things, but are urging prevention of dental disorders as a panacea for lean bank accounts!! Now, Dr. this is not criticism. It is merely igving a view not generally taken. Medicine and dentistry are radically different; nevertheless, there is much the dental profession could unselfishly do for the public good that can be profitable, thrashed out, and brought out, and if my comment tends in any wise to this end, I shall be very glad. We are in perfect agreement as to the vital points, however much we may differ as to details.

